# Supplementary material for: MetaRibo-Seq measures translation in microbiomes
Source: Nat Commun. 2020 Jun 29;11:3268. doi: 10.1038/s41467-020-17081-z (PMC7324362; doi:10.1038/s41467-020-17081-z)
Supplement: Supplementary file 10 — Supplementary Data 7 [file 41467_2020_17081_MOESM10_ESM.zip › File2/Confidence_High_Taxonomy/208903_out.krona.html]

Javascript must be enabled to view this page.

members
magnitude
magnitudeUnassigned
count
unassigned
taxon
rank

208903\_out

3

superkingdom
3
2

2
phylum
1239

186801
2
class

186802
order
2

186803
2
family

39491

SRS043701\_contig\_number\_11505
1
species

1
genus
28050

28052
species
1

SRS098827\_contig\_number\_12022

1798710
1
phylum

1906119
1
order


SRS147377\_contig\_number\_contig-100\_3.84990
species
1
1916232
